# Serum Levels of Joining Chain-Containing IgA1 Are Not Elevated in Patients with IgA Nephropathy

**DOI:** 10.1155/2019/9802839

**Published:** 2019-07-02

**Authors:** Guanhong Li, Xiaoyan Wang, Zhe Yang, Qing Zhao, Yubing Wen, Xuemei Li, Ruitong Gao

**Affiliations:** ^1^Division of Nephrology, Department of Internal Medicine, Peking Union Medical College Hospital, Chinese Academy of Medical Sciences and Peking Union Medical College, Beijing 100730, China; ^2^Department of Clinical Laboratory, Key Laboratory of Cancer Prevention and Therapy, National Clinical Research Center of Cancer, Tianjin Medical University Cancer Institute and Hospital, Tianjin 300060, China; ^3^Department of Immunology, School of Basic Medicine, Institute of Basic Medical Sciences, Chinese Academy of Medical Sciences and Peking Union Medical College, Beijing 100005, China

## Abstract

**Background:**

It has been suggested that mesangial IgA deposits are dimeric or polymeric in IgA nephropathy (IgAN). However, evidence concerning the molecular form of serum IgA in IgAN is controversial. And there is no direct evidence that the serum levels of joining chain- (J chain-) containing IgA (J-IgA) are elevated in IgAN. In this study, we aimed to measure serum J-IgA and glomerular J chain deposition with anti-J chain monoclonal antibody in IgAN.

**Methods:**

BALB/c mice were immunized with human J chain-GST recombinant peptide to obtain anti-J chain monoclonal antibody. The levels of serum total IgA and J-IgA were measured by sandwich enzyme-linked immunosorbent assay in 115 patients with IgAN and 117 healthy volunteers. J chain deposition in kidney specimens was analyzed by immunohistochemistry staining.

**Results:**

Serum levels of total IgA1 were elevated in IgAN patients compared to healthy subjects. However, serum levels of IgA, J-IgA, and J chain-containing IgA1 (J-IgA1), the J-IgA to total IgA ratio, and the J-IgA1 to total IgA1 ratio were not significantly different between IgAN patients and healthy subjects. Western blot analysis and gel filtration analysis using purified IgA1 also showed that the proportion of J chain-containing polymeric IgA1 was lower in IgAN patients compared to healthy subjects. No correlation was found between serum J-IgA or J-IgA1 and clinical features in IgAN. Immunohistochemistry analysis showed that glomerular J chain was positive in 12 IgAN patients (57.1%). The values of the J-IgA to IgA ratio and J-IgA1 to IgA ratio were significantly higher in IgAN patients with glomerular J chain deposition than those without. However, the serum levels of J-IgA and J-IgA1 and the J-IgA1 to IgA1 ratio were not significantly higher in two subgroups.

**Conclusions:**

Although serum levels of total IgA1 were elevated in IgAN, the serum levels of J-IgA1 were not elevated. And serum J-IgA, serum J-IgA1, and J chain deposition were not correlated with disease severity in IgAN.

## 1. Introduction

IgA nephropathy (IgAN) is characterized by the presence of IgA deposits in the mesangium and is the most common primary glomerular disease in the world [1]. More than one-third of patients with IgAN could progress to end-stage renal failure after 20–25 years, eventually requiring renal replacement therapy [2, 3]. Increasing evidence has suggested that IgAN is a systemic disease and the kidney itself is an innocent bystander. Studies have demonstrated that IgAN patients exhibit recurrent IgA deposition after renal transplantation [4–6] and non-IgAN patients who receive kidney graft from IgAN donors exhibit clearance of IgA deposits after several weeks [7]. However, the mechanism leading to renal IgA deposition is still unknown.

Although the pathogenesis of IgAN is still under investigation, researches from the past decades led to an agreement that the IgA deposits in the kidney are in dimeric or polymeric forms [8–10], revealing that those deposited IgA contain a small disulfide-linked polypeptide of 15 kDa known as the joining chain (J chain). It is generally agreed that J chain regulates the multimerization of IgA, forming secretory IgA (sIgA) and polymeric IgA (pIgA) [11–13]. J chain is also required for the transportation of sIgA across the mucosal epithelium, preventing attachment of bacteria and viruses to mucous membranes [13]. It is well known that the classic manifestation of IgAN is episodic hematuria with or without proteinuria following mucosal infection. It is possible that mucosal immunity dysregulation in IgAN might lead to the increased synthesis of sIgA which is transported across the mucosa with the help of J chain and thus reaches the circulation and deposit in mesangium.

Several investigations have revealed that the serum levels of sIgA and pIgA are elevated in IgAN [14–17]. However, there have been contradictory observations. van der Boog et al. found that pIgA concentrations relative to total IgA were significantly lower in sera of patients with IgAN [18]. In addition, Czerkinsky et al. reported that the circulating immune complexes from IgAN patients contained substantial amounts of monomeric IgA1 after acid dissociation [19]. Thus, the molecular form of serum IgA in IgAN remains controversial.

We obtained a novel monoclonal antibody (mAb) against J chain. In this study, we aimed to confirm if the concentrations of serum J-IgA and J-IgA1 in IgAN are elevated. And we also analyzed glomerular J chain deposition with anti-J chain mAb in IgAN.

## 2. Materials and Methods

### 2.1. Patients and Normal Subjects

We enrolled 115 patients with IgAN (IgAN group) from the Division of Nephrology, Department of Internal Medicine, Peking Union Medical College Hospital. One hundred and seventeen healthy volunteers (normal control group (NC group)) were all healthy blood donors who had no known kidney diseases enrolled from the Sanhe Pediatric Hospital and Peking Union Medical College Hospital. The histologic diagnosis of IgAN was based upon the demonstration of mesangioproliferative changes on light microscopy and the concomitant presence of predominant or codominant mesangial deposition of IgA. All participants gave informed consent to the study, which was performed in accordance with the Declaration of Helsinki and had received Local Ethical Committee approval.

### 2.2. Serum Samples

Blood samples from patients were collected after an overnight fast. All samples were allowed to clot at room temperature for 30 mins and centrifuged at 3000 rmp/min for 10 min to remove cellular components. Serum samples were kept at -80°C until use. Blood samples from healthy subjects were also collected and prepared in the same manner as for patients.

### 2.3. Clinical Parameters

The demographic and clinical parameters of patients, including age, gender, serum creatinine, and 24-hour urinary protein excretion (24hUPro), were obtained at the same time as that of sample collection. The estimated glomerular filtration rate (eGFR) was calculated using the Chronic Kidney Disease Epidemiology Collaboration (CKD-EPI) equation [20].

### 2.4. Generation of anti-J Chain Monoclonal Antibody

As the antigen, J chain-GST recombinant peptide IGJ-pGEX-4T-1 (NCBI ID for J chain sequence: NM_144646) was synthesized and purified. After three times of Freund adjuvant-conjugated antigen peptide administration to immunize BALB/c mice, candidate hybridomas were established from splenocytes (Absea Biotechnology Ltd., Beijing, China). Hybridomas that produce anti-J chain monoclonal antibodies were selected by dot blot with the pIgA purified from multiple myeloma patients and saliva that contains J chain-sIgA by Western Blot.

### 2.5. Measuring Serum Biomarkers

#### 2.5.1. Measurement of Serum Total IgA and IgA1

To measure serum total IgA, ninety-six-well microtiter plates were coated with anti-IgA mAb (Absea Biotechnology Ltd., Beijing, China) in carbonate-bicarbonate buffer, pH 9.6, overnight at 4°C. The plates were washed three times with phosphate-buffered saline (PBS) containing 0.05% Tween 20 (PBST) and then blocked with 200 *μ*L/well of 2% bovine serum albumin (BSA) for 2 h at room temperature. The serum samples were diluted 1 : 6000, added, and incubated for 1 h at room temperature. Then, the plates were washed and horse radish peroxidase- (HRP-) conjugated anti-human IgA mAb (Absea Biotechnology Ltd., Beijing, China) was added and incubated for 1 h at room temperature. After washing, the color was developed using ABTS (AMRESCO, Solon, USA) as a substrate and the absorbance was measured at 405 nm with a microplate reader (BioTek Synergy 4).

To measure serum IgA1, anti-IgA mAb (Absea Biotechnology Ltd., Beijing, China) was coated. After washing, the serum samples were diluted 1 : 20000, added, and incubated for 1 h at room temperature. Then, the plates were washed and anti-IgA1 mAb (AbD Serotec, Kidlington, UK) was added in 1 : 1000 dilution and incubated for 1 h at room temperature. The color was developed using ABTS as a substrate.

#### 2.5.2. Measurement of J Chain-Containing IgA (J-IgA) and J Chain-Containing IgA1 (J-IgA1)

To measure serum J-IgA, ninety-six-well microtiter plates were coated with mouse anti-J chain mAb overnight at 4°C. After blocking, the serum samples were added and incubated for 2 h at room temperature. After washing, HRP-conjugated anti-human IgA mAb was added and incubated for 1 h at room temperature. The color was developed using ABTS as a substrate.

To measure serum J-IgA1, anti-IgA1 mAb diluted 1 : 1000 in PBS was added and incubated for 1 h at room temperature. The color was developed using ABTS as a substrate, and the absorbance was measured at 450 nm with a microplate reader (BioTek Synergy 4, Winooski, VT, USA).

#### 2.5.3. Purification of IgA1 Using a Jacalin Column

4 mL serum samples from IgAN patients (a mixture of 10 serum samples from IgAN patients) or 4 mL normal control serum (a mixture of 10 serum samples from healthy subjects) was loaded onto a jacalin column [21, 22] (Pierce, Rockford, USA) at 4°C. After being washed with five-column volumes of PBS, the bound IgA1 was eluted with 0.1 M *α*-D-galactose in PBS.

#### 2.5.4. Western Blot Analysis

Purified IgA1 was run on 5-18% SDS-PAGE. The membrane with the molecular weight less than 46 kDa was incubated with anti-J chain antibody, with the HRP-conjugated goat anti-mouse IgG (MilliporeSigma, Darmstadt, German) as the secondary antibody. The membrane with the molecular weight greater than 46 kDa was incubated with HRP-conjugated anti-human IgA mAb (Absea Biotechnology Ltd., Beijing, China) for 1 h at room temperature. Then, the membranes were washed and the bands were visualized by enhanced chemiluminescence (Pierce, Rockford, USA).

#### 2.5.5. High-Performance Liquid Chromatography (HPLC) Analysis

A Viscotek P4000 column (Malvern Instruments Ltd., Malvern, UK) was balanced with PBS until it reached a smooth baseline. Then, 100 *μ*L of IgA1 purified from the jacalin column was loaded at a flow rate of 0.5 mL/min. Fractions of 50 *μ*L each were collected, and absorbance was monitored at 280 nm. Then, the collected fractions were detected for J chain by enzyme-linked immunosorbent assay (ELISA).

### 2.6. Immunohistochemistry and Immunofluorescence Staining

For each patient, the diagnosis of IgAN was based on histologic assessment of renal biopsy tissue with hematoxylin and eosin, periodic acid Schiff and Masson's trichrome for light microscopy, and staining with antibodies against IgA, IgG, IgM, C1q, C3, C4, and fibrinogen for immunofluorescence. The intensity of immunofluorescence was graded as (-), trace (±), (+), (++), (+++), and (++++). IgAN was defined by the presence of at least 1+ (range, 0–4) IgA mesangial deposits as dominant or codominant immunoglobulins on immunofluorescence microscopy performed on frozen tissue. Immunohistochemistry analysis was carried out according to conventional methods. In brief, tissue slices were incubated with mouse anti-J chain mAb overnight at 4°C. Then, the HRP-conjugated anti-mouse IgG mAb (MilliporeSigma, Darmstadt, German) was used as a secondary antibody. The color was visualized and developed using DAB (ZSGB-BIO) as a substrate.

### 2.7. Statistical Analyses

Statistical calculations were performed using SPSS software for Windows, version 20.0 (SPSS Inc., Chicago, IL) and GraphPad Prism 7.0 (GraphPad Software Inc., San Diego, CA). Data are presented as medians (interquartile range) or frequency in percent according to the types of variables. For nonnormal distribution parameters, the Mann–Whitney *U*-test was used for statistical comparisons between two groups. Bivariate correlation analyses were performed using Spearman's correlation analysis. *P* < 0.05 was considered significant.

## 3. Results

### 3.1. Anti-J Chain Monoclonal Antibody Recognizes J Chain

**Figure 1:**
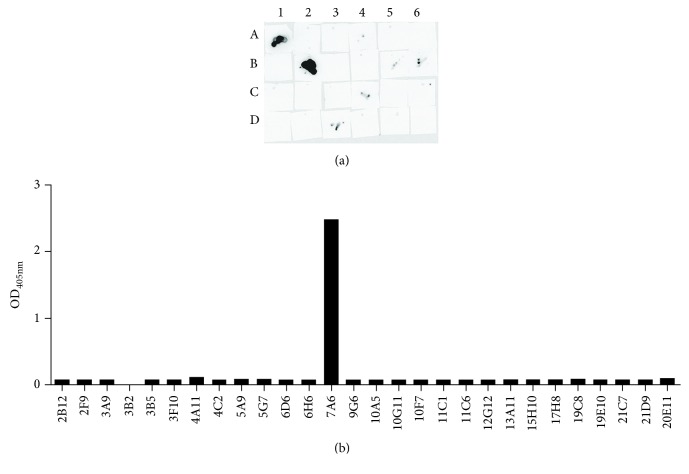
Selecting reliable and specific anti-J chain monoclonal antibodies to detect J chain. (a) Dot blot was performed to confirm the reactivity of anti-J chain monoclonal antibodies to pIgA purified from multiple myeloma patients. 7A6 has the best reactivity among 28 anti-J chain monoclonal antibodies. A1-A6: 2F9, 2B12, 3B2, 5G7, 3A9, 3B5; B1-B6: 6H6, 7A6, 10A5, 11C6, 19C8, and 20E11; C1-C6: 4A11, 5A9, 6D5, 9G6, 10F7, and 11C1; D1-D6: 10D11, 13A11, 15H10, 17H8, 19E10, and 21C7; (b) a sandwich ELISA for detecting the reactivity of anti-J chain to saliva which contains J chain-sIgA was performed. Anti-IgA antibody was immobilized on 96-well ELISA plates. After blocking, saliva samples were added and incubated for 2 h at room temperature. After washing, hybridoma cell culture supernatant was incubated. Plates were washed and incubated with HRP-conjugated goat anti-mouse antibody. The color was developed and the absorbance was measured at 405 nm. J chain: joining chain; pIgA: polymeric IgA; sIgA: secretory IgA; ELISA: enzyme-linked immunosorbent assay; HRP: horse radish peroxidase.

To obtain anti-J chain monoclonal antibodies, J chain-GST recombinant peptide IGJ-pGEX-4T-1 was synthesized as antigen to immunize BALB/c mice. Several hybridoma clones were generated. Hybridomas that produce anti-J chain mAbs were selected by ELISA with the recombinant antigen peptide (data not shown) and by dot blot with pIgA purified from multiple myeloma patients (Figure 1(a)). Furthermore, ELISA was performed with saliva that contains J chain-sIgA to confirm the reactivity and specificity of anti-J chain monoclonal antibodies (Figure 1(b)). We observed that 7A6 has the best reactivity to recombinant J chain peptide and saliva.

### 3.2. Demographic and Clinical Features in the IgAN Patients and Healthy Subjects

In the present study, 115 patients with IgAN (mean age, 35 (30–43) years; male/female ratio, 58/57) and 117 healthy subjects (mean age, 37 (27–48) years; male/female ratio, 46/71) were involved. The main demographic and clinical parameters were summarized in Table 1. The average levels of 24hUPro and eGFR were 0.63 (0.26–1.44) g/24 h and 93.31 (70.90-123.10) mL/min·1.73 m^2^, respectively.

### 3.3. Concentrations of Serum Total IgA and J-IgA in Patients with IgAN and Controls

**Figure 2:**
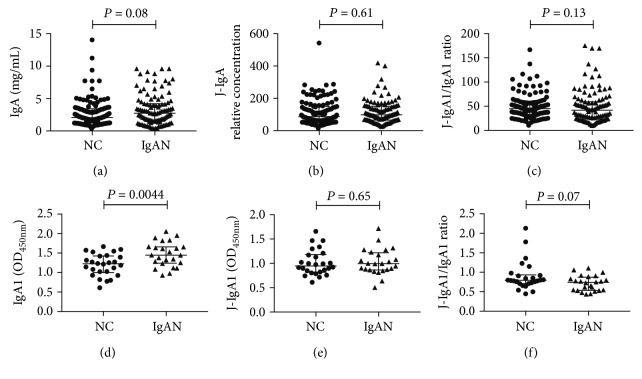
Comparison of serum IgA in patients with IgA nephropathy and heathy subjects. J-IgA: joining chain-containing IgA; J-IgA1: joining chain-containing IgA1; IgAN: IgA nephropathy; NC: normal control. Statistically significant differences were tested with Mann–Whitney *U*-test.

ELISA was performed to measure serum J-IgA with anti-J chain mAb 7A6. The levels of serum total IgA (IgAN: 2.73 (1.49-4.20) vs. NC: 2.05 (1.20-3.41), *P* = 0.08) tended to be higher in the IgAN group compared to the NC group, but the *P* value was not significant. The levels of serum J-IgA (IgAN: 98.30 (65.78-151.30) vs. NC: 87.10 (61.22-150.30), *P* = 0.61) were not significantly different between IgAN patients and healthy subjects (Figures 2(a) and 2(b)). And the value of the J-IgA to total IgA ratio was also not significantly different between two groups (IgAN: 41.95 (23.63-59.31) vs. NC: 44.92 (33.51-57.88), *P* = 0.13) (Figure 2(c)).

### 3.4. Concentrations of Serum Total IgA1 and J-IgA1 in Patients with IgAN and Controls

We measured the serum levels of total IgA1 and J-IgA1 in IgAN patients (*n* = 24) and healthy subjects (*n* = 26). The levels of serum total IgA1 were significantly higher in the IgAN group than those in the NC group (IgAN: 1.45 (1.24-1.66) OD_450 nm_ vs. NC: 1.43 (1.23-1.67) OD_450 nm_, *P* = 0.0044) (Figure 2(d)). However, no significant difference was found in the serum levels of J-IgA1 between the IgAN group and NC group (IgAN: 1.00 (0.87-1.22) OD_450nm_ vs. NC: 0.95 (0.82-1.18) OD_450nm_, *P* = 0.65). While the value of the J-IgA1 to total IgA1 ratio tended to be lower in the IgAN group compared to the NC group, the *P* value was not significant (IgAN: 0.74 (0.54-0.88) vs. NC: 0.80 (0.71-0.94), *P* = 0.07) (Figures 2(e) and 2(f)).

**Figure 3:**
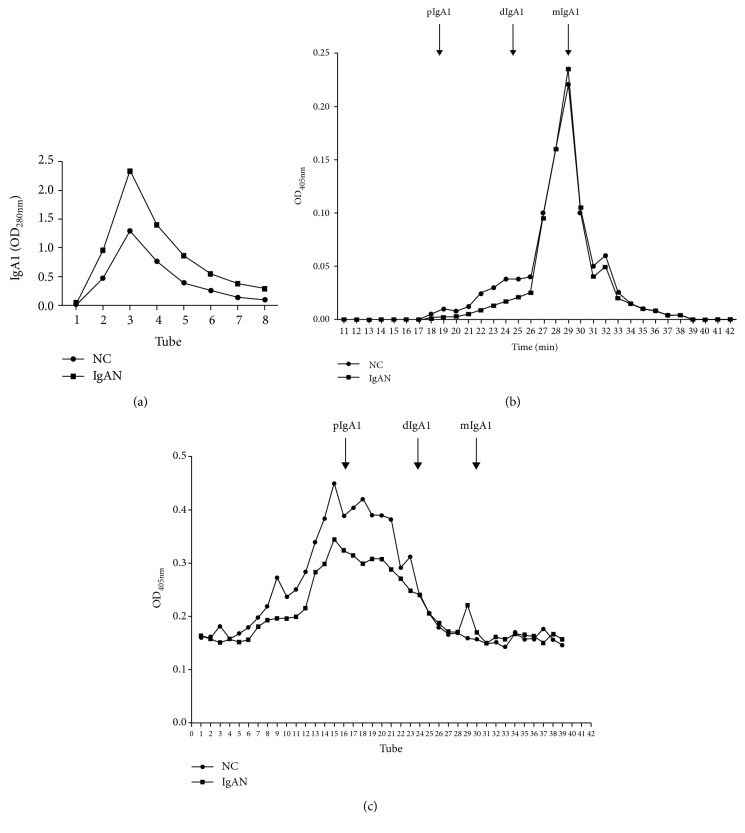
Gel filtration analysis of IgA1 purified from patients with IgA nephropathy and healthy subjects. pIgA1: polymeric IgA1; dIgA1: dimeric IgA1; mIgA1: monomeric IgA1; IgAN: IgA nephropathy; NC: normal control.

**Figure 4:**
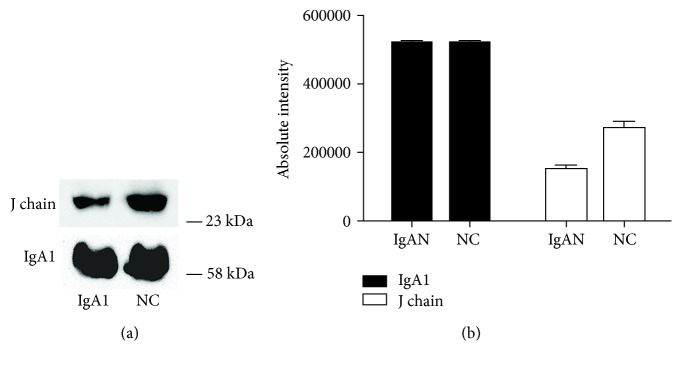
Western blot analysis of IgA1 purified from patients with IgA nephropathy and healthy subjects. J-IgA: joining chain-containing IgA; IgAN: IgA nephropathy.

To further confirm the difference in the J-IgA1 to total IgA1 ratio in IgAN patients and healthy subjects, we purified IgA1 from 4 mL mixed serum samples of IgAN patients (*n* = 10) or 4 mL mixed serum samples of healthy subjects (*n* = 10) through a jacalin column. The amount of IgA1 purified from IgAN patients was significantly greater than that from the healthy subjects (Figure 3(a)). We performed gel filtration analysis using IgA1 purified from jacalin columns. An equal amount of IgA1 from two groups was loaded. It was shown that higher levels of monomeric IgA1 (mIgA1) were eluted in the IgAN group, while lower levels of dIgA1 and pIgA1 were shown in the IgAN group than those in the NC group (Figure 3(b)). We analyzed the percentage of mIgA1, dIgA1, and pIgA1 by calculating the area under each chromatographic peak in Figure 3(b). It showed that the levels of dIgA1 and pIgA1 were lower in the IgAN group than those in the NC group (Table 2). And we further confirmed the levels of J chain-containing dIgA and pIgA in the column effluent with anti-J chain mAb by ELISA. Lower levels of J chain-containing dIgA and J chain-containing pIgA1 were shown in the IgAN group than those in the NC group (Figure 3(c)). Western blot analysis also showed that less J chain was found in the IgAN group than that in the NC group when a similar amount of IgA1 from these two groups was analyzed (Figure 4).

### 3.5. Correlations between Serum Levels of J-IgA1 and Clinical Features of Patients with IgAN

Spearman's analyses showed that serum levels of J-IgA1 and J-IgA in IgAN patients did not significantly correlate with 24hUpro and renal function (Table 3).

### 3.6. J Chain Staining on Kidney Specimens

**Figure 5:**
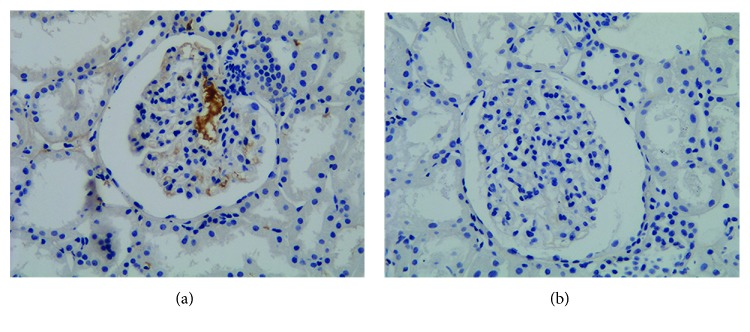
Renal tissues from patients with IgAN were stained for the presence of J chain. (a) A patient with positive J chain staining; (b) a representative patient with negative J chain staining. Original magnification: ×200 in (a, b). IgAN: IgA nephropathy; J chain: joining chain.

**Figure 6:**
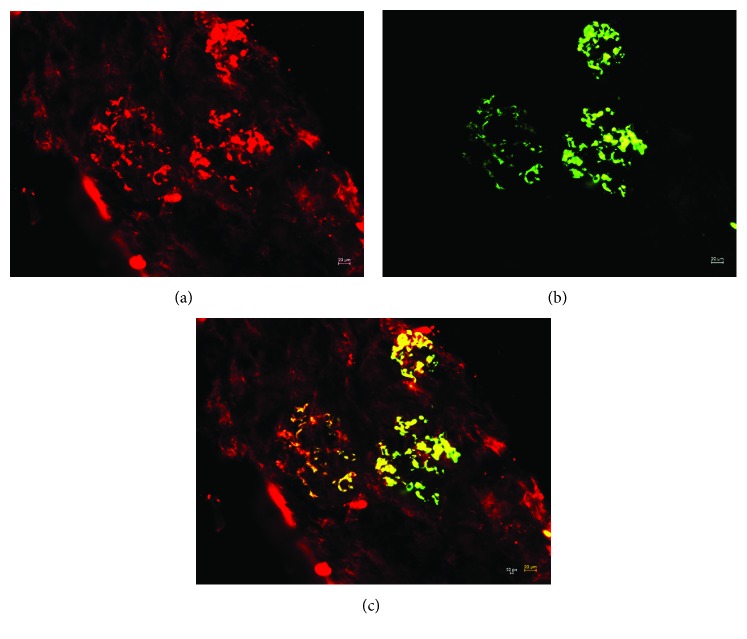
Renal tissues from patients with IgAN were double-stained for the presence of J chain and IgA. (a) J chain staining; (b) IgA staining; (c) merged image. Original magnification: ×100 in (a–c).

**Figure 7:**
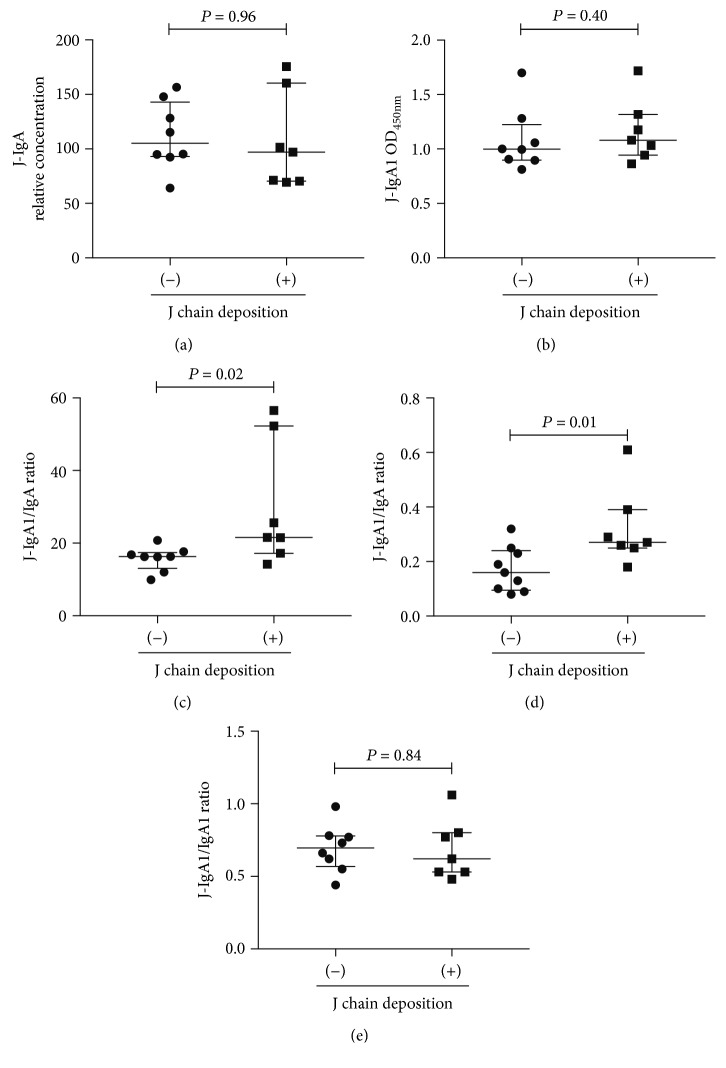
Comparison of serum J-IgA and J-IgA1 in IgAN patients with or without J chain deposition in the kidney. IgAN: IgA nephropathy; J chain: joining chain; J-IgA: J chain-containing IgA; J-IgA1: J chain-containing IgA1.

We performed immunohistochemistry staining of J chain on kidney biopsy samples of 21 IgAN patients. Glomerular J chain was positive in 12 patients (57.1%). The profile of immunofluorescence and immunohistochemistry staining of these 12 IgAN patients was summarized in Table 4 and Figure 5. Confocal immunofluorescence was performed on the kidney slides of an IgAN patient with anti-J chain mAb and anti-IgA antibody. The J chain deposition was distributed similarly with the deposition of IgA (Figure 6). The values of the serum J-IgA to IgA ratio and J-IgA1 to IgA ratio were significantly higher in J chain-positive patients. However, serum levels of J-IgA, the J-IgA to IgA ratio, and the J-IgA1 to IgA1 ratio were not significantly different in two subgroups (Figure 7). And the levels of serum J-IgA1, serum J-IgA, 24hUpro, and renal function did not significantly correlate with the percentage of J chain deposition (Table 5).

Immunohistochemistry staining of J chain was also performed on kidney biopsy samples of 5 lupus nephritis patients. However, no glomerular J chain deposition was found on the kidney specimens of these patients (Supplementary Table 1 and Supplementary Figure 1).

## 4. Discussion

According to previous studies, it has been suggested that IgA deposits in the kidneys of patients with IgAN are dimeric or polymeric IgA [8–10], indicating that the J chain-linked pIgA might be the source of pathologic immunoglobulin in IgAN. Although the origin of the pathologic IgA1 deposited in renal mesangium has been a subject of debate, it has been widely accepted that these IgA immune deposits in the kidneys are likely derived from the circulation. Some previous studies showed that levels of serum macromolecular IgA were elevated in IgAN patients [14–17, 24]. These elevated macromolecular IgA has been reported as polymeric and J chain associated; the levels of J-IgA in IgAN remain unknown.

In this study, we aimed to further confirm the following: (1) whether the levels of serum J chain-containing pIgA are elevated and (2) the proportion of J chain is positive in the kidneys of IgAN patients. To the best of our knowledge, we obtained the anti-J chain mAb for the first time. We used this anti-J chain mAb to measure the levels of serum J-IgA and J-IgA1 in patients with IgAN and healthy subjects. Our data showed that the levels of serum total IgA1 were elevated in IgAN patients. However, surprisingly, the levels of serum J-IgA and J-IgA1 were not significantly elevated in IgAN patients compared to healthy subjects, which was confirmed by Western blot and gel filtration analysis. And the serum J-IgA and J-IgA1 did not correlate with clinical severity in IgAN patients. The data in the present study are inconsistent with previous reports. Previous studies reported that circulating levels of secretory IgA and polymeric IgA were found to be elevated in IgAN patients compared to healthy subjects [14–17, 24]. However, the serum macromolecular IgA in these studies were measured by secretory component-binding enzyme immunoassay, radioimmunoassay, ELISA with an antibody specific for secretory component, or analyzing the size fractionation of IgA. But no studies tried to detect the J chain-linked IgA with a monoclonal antibody. And some other evidence concerning the molecular form of serum IgA in IgAN are contradictory [18, 25]. Peterman et al. found that the levels of monomeric IgAl were elevated in IgAN patients with normal renal function [25]. And van der Boog et al. did not find significant correlation between serum concentrations of pIgA and clinical parameters of the disease [18].

Earlier studies use antiserum to J chain to detect J chain in kidney specimens of IgAN patients [8, 9, 26]. And some other studies detected IgA in material eluted from kidney sections to define the molecular form of the pathogenic IgA [26, 27]. However, these studies using the methods described above might be of limited value. Firstly, there is a lack of specialty in detecting the presence of J chain in mesangial deposits by anti-J chain sera. Secondly, measuring the elution from the kidney is not reliable enough due to the complexed elution preprocess which may cause a complicated result. In this study, we detected J chain in the kidney specimens of IgAN patients with an anti-J chain mAb produced by our laboratory, which is more specific and reliable. We found that glomerular J chain was positive in over half of the IgAN patients. And the values of the serum J-IgA to IgA ratio and J-IgA1 to IgA ratio were significantly higher in IgAN patients with J chain deposition than those without. It suggests that these IgA deposits in IgAN patients positive for glomerular J chain may be an IgA1 subclass.

It is well known that IgA immune complexes play an important role in the pathogenesis of IgAN [28–30]. O-Glycans of IgA1 in IgAN are characterized by the presence of N-acetylgalactosamine within galactose. IgA1-containing immune complexes are formed when IgG and IgA autoantibodies recognize the reduced glycosylated IgA1 in the circulation [30–34]. Whether IgA can form polymers to become macromolecular IgA in the absence of the J chain is still a question. Satake et al. found that dominant immune complexes in glomeruli consisted of the following: (1) IgA1-IgG and complements, (2) pIgA1 and complements, and (3) monomeric IgA1-IgA or aggregated monomeric IgA1 [35]. It suggested that the IgA-containing complexes are in a composite form. But they did not analyze the J chain-containing IgA in their study. Researchers discovered that polymeric IgA1 could exist in the bodies of J chain knockout mice, suggesting that J chain is not required for IgA dimerization or polymerization [36]. Kolka et al. also found that J chain-deficient mice showed increased polymeric serum IgA [37]. It is possible that part of the polymeric IgA contains J chain, while some monomeric IgA aggregates together to become macromolecular IgA without the help of J chain.

However, there are some limitations to this study. Firstly, we did not detect the glycosylation of J-IgA1 or perform a glycosylation analysis for the purified IgA1. Whether the IgA of IgAN patients is prone to self-aggregate due to poor glycosylation requires further study. Secondly, the number of kidney specimens for detecting J chain in the present study was limited.

In summary, our data demonstrated that the levels of serum J-IgA and J-IgA1 were not increased in all patients with IgAN and did not correlate with urine protein and renal function. Over half of the IgAN patients presented with renal J chain deposition. The levels of J-IgA/total IgA and J-IgA1/total IgA were elevated in patients with IgAN with glomerular J chain deposition.

## Figures and Tables

**Table 1:** Demographic and clinical features of patients with IgA nephropathy and healthy subjects.

Characteristics	IgAN	NC
Patient number	115	117
Mean age (yr)	35 (30-43)	37 (27-48)
Male, *n* (%)	58 (50.4)	46 (39.3)
24-hour urine protein excretion (g/24 h)	0.63 (0.26-1.44)	
<0.3	32 (27.8%)	
0.3-0.99	44 (38.3%)	
1.0-2.99	23 (20.0%)	
≥3	16 (13.9%)	
Serum creatinine (mg/dL)	1.00 (0.79-1.36)	
eGFR (mL/min·1.73 m^2^)^a^	93.31 (70.90-123.10)	
CKD stages^b^, *n* (%)		
1	63 (54.8)	
2	31 (27.0)	
3a	12 (10.4)	
3b	6 (5.2)	
4	2 (1.7)	
5	1 (0.9)	

Data are presented as median (interquartile range) or frequency in percentage. ^a^eGFR was calculated according to the Chronic Kidney Disease Epidemiology Collaboration (CKD-EPI) equation [20]. ^b^CKD stages 1–5 were divided by eGFR ≥ 90 (G1), 60–89 (G2), 45–59 (G3a), 30–44 (G3b), 15–29 (G4), and <15 (G5) mL/min/1.73 m^2^, respectively, according to the new KDIGO (Kidney Disease: Improving Global Outcomes) classification [23].

**Table 2:** Comparison of the percentage of mIgA1, dIgA1, and pIgA1 in purified IgA1 between patients with IgA nephropathy and healthy subjects.

	pIgA1 (%)	dIgA1 (%)	mIgA1(%)	Small fragments (%)
NC	1.36	10.67	75.77	12.20
IgAN	0.20	4.96	84.33	10.52

IgAN: IgA nephropathy; NC: normal control; pIgA1: polymeric IgA1; dIgA1: dimeric IgA1; mIgA1: monomeric IgA1.

**Table 3:** Correlation between serum J-IgA1 and clinical factors in patients with IgA nephropathy.

Variables	J-IgA		J-IgA/IgA		IgA1		J-IgA1/IgA1	
	^a^Coefficient	*P* value	Coefficient	*P* value	Coefficient	*P* value	Coefficient	*P* value
24-hour urine protein excretion	0.13	0.20	-0.06	0.54	-0.03	0.89	-0.01	0.98
Serum creatinine	0.16	0.11	0.16	0.10	-0.10	0.97	0.17	0.30
eGFR^b^	-0.14	0.15	-0.16	0.11	0.07	0.74	-0.34	0.11

eGFR: estimated glomerular filtration rate. ^a^Spearman's correlation analysis unless otherwise specified. ^b^eGFR was calculated according to the Chronic Kidney Disease Epidemiology Collaboration (CKD-EPI) equation [20].

**Table 4:** Immunofluorescent and immunohistochemistry staining of immunoglobulins and J chain on kidney slides of patients with IgA nephropathy.

Patient number	Intensity/percentage of staining				
	IgA	IgM	IgG	C3	J chain (%)
1	+++	—	±	+	50.0 (6/12)
2	+++	—	—	++	100.0 (19/19)
3	+++~++++	+	±~+	++~+++	55.0 (11/20)
4	++	±	—	+	76.0 (19/25)
5	+++~++++	—	—	++	91.7 (11/12)
6	+++	—	—	±	77.8 (14/18)
7	++~+++	±	—	+	81.8 (18/22)
8	+++	+~++	—	+	100.0 (13/13)
9	+++~++++	—	—	++	66.7 (6/9)
10	++	—	—	±~+	76.5 (13/17)
11	++	—	—	—	83.3 (10/12)
12	+++	±	±	—	60.0 (9/15)

**Table 5:** Correlation between J chain deposition and clinical factors.

Variables	Coefficient	*P* value
J-IgA	0.42	0.18
J-IgA/IgA	0.49	0.11
J-IgA1	0.57	0.18
J-IgA1/IgA1	0.21	0.65
24-hour urine protein excretion	-0.14	0.66
Serum creatinine	-0.47	0.12
eGFR	0.31	0.34

J chain: joining chain; J-IgA: J chain-containing IgA; J-IgA1: J chain-containing IgA1; eGFR: estimated glomerular filtration rate. ^a^Spearman's correlation analysis unless otherwise specified. ^b^eGFR was calculated according to the Chronic Kidney Disease Epidemiology Collaboration (CKD-EPI) equation [20].

## Data Availability

The data used to support the findings of this study are available from the corresponding author upon request.

## Supplementary Materials

Supplementary Materials Supplementary Table 1: Immunofluorescent/immunohistochemistry staining of immunoglobulins and J chain on kidney specimens of patients with lupus nephritis.

Supplementary Materials Supplementary Figure 1: Renal tissues from a patient with lupus nephritis with negative J chain staining.

## References

[1] D'Amico G. (1987). The commonest glomerulonephritis in the world: IgA nephropathy. *QJM: An International Journal of Medicine*.

[2] Imai H., Miura N. (2012). A treatment dilemma in adult immunoglobulin A nephropathy: what is the appropriate target, preservation of kidney function or induction of clinical remission?. *Clinical and Experimental Nephrology*.

[3] D’Amico G. (2000). Natural history of idiopathic IgA nephropathy: role of clinical and histological prognostic factors. *American Journal of Kidney Diseases*.

[4] Berger J., Yaneva H., Nabarra B., Barbanel C. (1975). Recurrence of mesangial deposition of IgA after renal transplantation. *Kidney International*.

[5] Ji S., Liu M., Chen J. (2004). The fate of glomerular mesangial IgA deposition in the donated kidney after allograft transplantation. *Clinical Transplantation*.

[6] Ponticelli C., Glassock R. J. (2010). Posttransplant recurrence of primary glomerulonephritis. *Clinical Journal of the American Society of Nephrology*.

[7] Silva F. G., Chander P., Pirani C. L., Hardy M. A. (1982). Disappearance of glomerular mesangial IgA deposits after renal allograft transplantation. *Transplantation*.

[8] Komatsu N., Nagura H., Watanabe K., Nomoto Y., Kobayashi K. (1983). Mesangial deposition of J chain-linked polymeric IgA in IgA nephropathy. *Nephron*.

[9] Donini U., Casanova S., Zini N., Zucchelli P. (1983). The presence of J chain in mesangial immune deposits of IgA nephropathy. *Proceedings of the European Dialysis and Transplant Association European Dialysis and Transplant Association*.

[10] Oortwijn B. D., Rastaldi M. P., Roos A., Mattinzoli D., Daha M. R., van Kooten C. (2007). Demonstration of secretory IgA in kidneys of patients with IgA nephropathy. *Nephrology Dialysis Transplantation*.

[11] Brandtzaeg P. (1974). Presence of J chain in human immunocytes containing various immunoglobulin classes. *Nature*.

[12] Brandtzaeg P. (1975). Immunochemical studies on free and bound J chain of human IgA and IgM. *Scandinavian Journal of Immunology*.

[13] Castro C. D., Flajnik M. F. (2014). Putting J chain back on the map: how might its expression define plasma cell development?. *The Journal of Immunology*.

[14] Lozano L., García-Hoyo R., Egido J. (1987). IgA nephropathy: association of a history of macroscopic hematuria episodes with increased production of polymeric IgA. *Nephron*.

[15] Jones C. L., Powell H. R., Kincaid-Smith P., Roberton D. M. (1990). Polymeric IgA and immune complex concentrations in IgA-related renal disease. *Kidney International*.

[16] Trascasa M. L., Egido J., Sancho J., Hernando L. (1980). IgA glomerulonephritis (Berger’s disease): evidence of high serum levels of polymeric IgA. *Clinical and Experimental Immunology*.

[17] Zhang J. J., Xu L. X., Liu G., Zhao M. H., Wang H. Y. (2007). The level of serum secretory IgA of patients with IgA nephropathy is elevated and associated with pathological phenotypes. *Nephrology Dialysis Transplantation*.

[18] Van der Boog P. J. M., van Kooten C., van Seggelen A. (2004). An increased polymeric IgA level is not a prognostic marker for progressive IgA nephropathy. *Nephrology Dialysis Transplantation*.

[19] Czerkinsky C., Koopman W. J., Jackson S. (1986). Circulating immune complexes and immunoglobulin A rheumatoid factor in patients with mesangial immunoglobulin A nephropathies. *Journal of Clinical Investigation*.

[20] Wang J., Xie P., Huang J. M. (2016). The new Asian modified CKD-EPI equation leads to more accurate GFR estimation in Chinese patients with CKD. *International Urology and Nephrology*.

[21] Linossier M. T., Palle S., Berthoux F. (2003). Different glycosylation profile of serum IgA1 in IgA nephropathy according to the glomerular basement membrane thickness: normal versus thin. *American Journal of Kidney Diseases*.

[22] Leung J. C. K., Tang S. C. W., Lam M. F., Chan T. M., Lai K. N. (2001). Charge-dependent binding of polymeric IgA1 to human mesangial cells in IgA nephropathy. *Kidney International*.

[23] Work Group Membership (2013). Chapter 2: Definition, identification, and prediction of CKD progression. *Kidney International Supplements*.

[24] Tomino Y., Miura M., Suga T. (1984). Detection of polymeric IgA in sera from patients with IgA nephropathy determined by thin-layer gel filtration. *The Tokai Journal of Experimental and Clinical Medicine*.

[25] Peterman J. H., Julian B. A., Kirk K. A., Jackson S. (1991). Selective elevation of monomeric IgA1 in IgA nephropathy patients with normal renal function. *American Journal of Kidney Diseases*.

[26] Tomino Y., Sakai H., Miura M., Endoh M., Nomoto Y. (1982). Detection of polymeric IgA in glomeruli from patients with IgA nephropathy. *Clinical and Experimental Immunology*.

[27] Monteiro R. C., Halbwachs-Mecarelli L., Roque-Barreira M. C., Noel L. H., Berger J., Lesavre P. (1985). Charge and size of mesangial IgA in IgA nephropathy. *Kidney International*.

[28] Allen A. C., Harper S. J., Feehally J. (1995). Galactosylation of N- and O-linked carbohydrate moieties of IgA1 and IgG in IgA nephropathy. *Clinical & Experimental Immunology*.

[29] Moldoveanu Z., Wyatt R. J., Lee J. Y. (2007). Patients with IgA nephropathy have increased serum galactose-deficient IgA1 levels. *Kidney International*.

[30] Lai K. N. (2012). Pathogenesis of IgA nephropathy. *Nature Reviews Nephrology*.

[31] Novak J., Julian B. A., Mestecky J., Renfrow M. B. (2012). Glycosylation of IgA1 and pathogenesis of IgA nephropathy. *Seminars in Immunopathology*.

[32] Suzuki H., Fan R., Zhang Z. (2009). Aberrantly glycosylated IgA1 in IgA nephropathy patients is recognized by IgG antibodies with restricted heterogeneity. *Journal of Clinical Investigation*.

[33] Suzuki H., Kiryluk K., Novak J. (2011). The pathophysiology of IgA nephropathy. *Journal of the American Society of Nephrology*.

[34] Tomana M., Novak J., Julian B. A., Matousovic K., Konecny K., Mestecky J. (1999). Circulating immune complexes in IgA nephropathy consist of IgA1 with galactose-deficient hinge region and antiglycan antibodies. *Journal of Clinical Investigation*.

[35] Satake K., Shimizu Y., Sasaki Y. (2014). Serum under-O-glycosylated IgA1 level is not correlated with glomerular IgA deposition based upon heterogeneity in the composition of immune complexes in IgA nephropathy. *BMC Nephrology*.

[36] Hendrickson B. A., Conner D. A., Ladd D. J. (1995). Altered hepatic transport of immunoglobulin A in mice lacking the J chain. *Journal of Experimental Medicine*.

[37] Kolka R., Valdimarsson H., Bodvarsson M., Hardarson S., Jonsson T. (2013). Defective immunoglobulin A (IgA) glycosylation and IgA deposits in patients with IgA nephropathy. *APMIS*.

