# Supplementary material for: Serum Levels of Joining Chain-Containing IgA1 Are Not Elevated in Patients with IgA Nephropathy
Source: Dis Markers. 2019 Jul 2;2019:9802839. doi: 10.1155/2019/9802839 (PMC6636472; doi:10.1155/2019/9802839)
Supplement: Supplementary Materials — Supplementary Table 1: Immunofluorescent/immunohistochemistry staining of immunoglobulins and J chain on kidney specimens of patients with lupus nephritis. [file 9802839.f1.docx]

| **Supplementary Table 1.** Immunofluorescent/immunohistochemistry staining of immunoglobulins and J chain on kidney specimens of patients with lupus nephritis. | | | | | | | |
| --- | --- | --- | --- | --- | --- | --- | --- |
|  |  |  |  |  |  |  |  |
| Patient number |  |  | Intensity/percentage of staining | | | |  |
|  | Gender | Age (years) | IgA | IgM | IgG | C3 | J chain (%) |
| 1 | Female | 27 | ++ | +～++ | ++ | ++～+++ | 0(0/19) |
| 2 | Female | 30 | + | + | +～++ | + | 0(0/12) |
| 3 | Female | 45 | + | - | ++ | +～++ | 0(0/13) |
| 4 | Male | 38 | ++ | + | ± | ±～+ | 0(0/4) |
| 5 | Male | 36 | + | ±～+ | ++ | + | 0(0/11) |
