# Supplementary material for: Serum Levels of Joining Chain-Containing IgA1 Are Not Elevated in Patients with IgA Nephropathy
Source: Dis Markers. 2019 Jul 2;2019:9802839. doi: 10.1155/2019/9802839 (PMC6636472; doi:10.1155/2019/9802839)
Supplement: Supplementary Materials — Supplementary Figure 1: Renal tissues from a patient with lupus nephritis with negative J chain staining. [file 9802839.f2.docx]

**
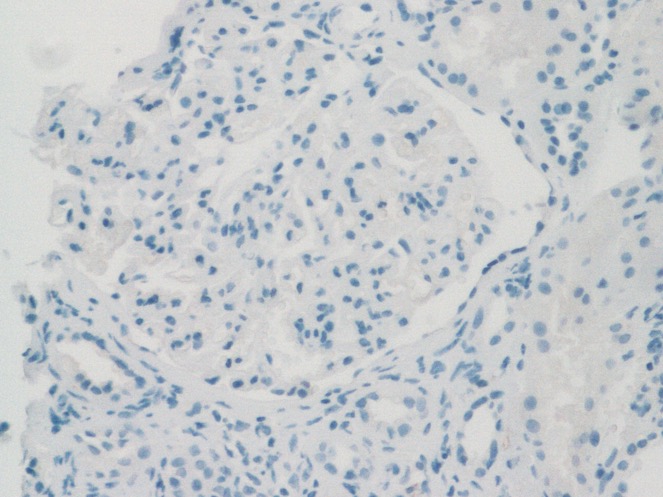
**

**Supplementary Figure 1. Renal tissues from a patient with lupus nephritis with negative J chain staining.** Original magnification：× 200
